# Vitamin D Receptor Gene Polymorphism and the Risk of Colorectal Cancer: A Nested Case-Control Study

**DOI:** 10.1371/journal.pone.0164648

**Published:** 2016-10-13

**Authors:** Sanjeev Budhathoki, Taiki Yamaji, Motoki Iwasaki, Norie Sawada, Taichi Shimazu, Shizuka Sasazuki, Teruhiko Yoshida, Shoichiro Tsugane

**Affiliations:** 1 Epidemiology and Prevention Group, Center for Public Health Sciences, National Cancer Center, Tokyo, Japan; 2 Division of Genetics, National Cancer Center Research Institute, Tokyo, Japan; National Health Research Institutes, TAIWAN

## Abstract

Epidemiological and experimental evidence suggest that vitamin D is protective against the risk of colorectal cancer. Polymorphisms in the gene encoding vitamin D receptor (VDR), which mediates most of the known cellular effects of vitamin D, have been suggested to alter this association. Here, using a tag SNP approach, we comprehensively evaluated the role of common genetic variants in *VDR* and their interaction with plasma vitamin D levels in relation to colorectal cancer risk in Japanese populations. A total of 356 colorectal cancer cases and 709 matched control subjects were selected from the participants of the Japan Public Health Center-based Prospective Cohort Study. Among these subjects, 29 *VDR* single nucleotide polymorphisms (SNPs) were selected and genotyped, and plasma vitamin D concentrations were measured. Conditional logistic regression models were used to estimate odds ratios (ORs) and 95% confidence intervals (CIs) of colorectal cancer, with adjustment for potential confounding factors. Among the results, eight *VDR* SNPs, namely rs2254210, rs1540339, rs2107301, rs11168267, rs11574113, rs731236, rs3847987 and rs11574143, the latter 5 of which were located in the 3′ region, were nominally associated with the risk of colorectal cancer (*P* = 0.01–0.048). Furthermore, of the above 5 3′ region SNPs, the inverse associations for 3 SNPs (rs11574113, rs3847987 and rs11574143) appeared to be evident only in those with high plasma vitamin D concentration. However, neither of these direct and suggestive interaction analysis associations was significant after multiple testing adjustment. Overall, the findings of this study provide only limited support for an association between common genetic variations in *VDR* and colorectal cancer risk in the Japanese population.

## Introduction

Since Garland and Garland’s 1980 report that the mortality rate of colon cancer was higher in regions that receive relatively low solar ultraviolet radiation, the protective role of vitamin D in colorectal carcinogenesis has attracted considerable interest [1, 2]. Vitamin D in the body is primarily derived from endogenous synthesis in the skin following exposure to solar ultraviolet radiation, and to a lesser extent from dietary sources. Vitamin D from these sources is converted to the circulating form 25-hydroxyvitamin D [25(OH)D] in the liver, which is further hydroxylated to the biologically active form 1,25-dihydroxyvitamin D [1,25(OH)_2_D] in the kidney and other tissues [3]. In experimental studies, 1,25(OH) _2_D has been shown to have anti-carcinogenic properties, including regulation of cellular proliferation and differentiation, induction of apoptosis, and inhibition of angiogenesis [2–4]. Although epidemiological studies of dietary vitamin D intake and colorectal cancer have generally shown a null to weakly inverse association, those which measured circulating 25(OH)D have fairly consistently demonstrated a significant inverse association [5, 6]. In fact, in our earlier analysis of food frequency questionnaire data, we did not observe any association between vitamin D intake and colorectal cancer risk in a population of over 70,000 adults, including 761 colorectal cancer cases [7]. By measuring circulating concentration in a subgroup of this population, however, we identified an inverse association between plasma vitamin D levels and rectal cancer, although such association was not apparent for colon cancer [8].

The cellular effects of vitamin D are mediated via binding of 1,25(OH) _2_D to vitamin D receptor (VDR), a member of the nuclear receptor superfamily which regulates the transcription of genes, including proto-oncogenes and tumor suppressor genes [3, 4, 9]. VDRs are expressed in a wide range of tissues, including normal and malignant colorectal tissue [10]. It has been hypothesized that common variations in the *VDR* gene may influence VDR expression and function, which in turn may lead to differential effects of vitamin D among individuals [9]. Although the *VDR* gene is large (>100 kb), and a number of single nucleotide polymorphisms (SNPs) have been discovered in it [11, 12], only a few comprehensive analyses have captured a large fraction of common SNPs in *VDR* in relation to colorectal cancer [13–15].

The group-specific component (*GC*) gene encodes the vitamin D-binding protein (VDBP), which plays a role in the binding and transport of vitamin D and its metabolites in circulation [16]. Two common variants in the *GC* gene, rs7041 and rs4588, lead to amino acid changes that result in different VDBP isotypes with altered affinity for vitamin D metabolites [17]. Several studies also showed that *GC* variants, including the two SNPs above, are significantly associated with 25(OH)D concentrations [18] and might therefore be of importance in the etiology of colorectal cancer.

Here, we aimed to evaluate the associations between common variation in the *VDR* gene and colorectal cancer risk using a comprehensive tag SNP approach. We also assessed the association between two common SNPs in the *GC* gene and colorectal cancer risk. Furthermore, because we observed no apparent association between plasma 25(OH)D and colorectal cancer risk in our previous study [8], we reevaluated this association in consideration of *VDR* polymorphism.

## Subjects and Methods

### Study population

The participants in the present analysis were taken from our earlier case-control study [8] nested within the Japan Public Health Center-based Prospective Cohort (JPHC) [7]. Details of the design and conduct of the JPHC study and nested-case control study have been described elsewhere [7, 8, 19]. In brief, the Japan Public Health Center-based Prospective Study (JPHC Study) was conducted in two cohorts, Cohort I initiated in 1990 and Cohort II in 1993. Cohort I enrolled 61,595 registered residents aged 40–59 years in five Public Health Center (PHC) areas across Japan, and Cohort II enrolled 78,825 registered residents aged 40–69 years in six PHC areas. In total, 68,721 men and 71,699 women were included in the JPHC study. At baseline, we informed all subjects orally and/or in writing about the details of the study, and asked them to answer the survey questionnaires. If a subject answered and returned the questionnaires, this was regarded as their consent to participate in this study. Following the baseline survey, we have since mailed the details of the study and related information several times to all study subjects, and have provided the opportunity to refuse participation in the study throughout the follow-up period. This consent procedure is consistent with the first ethical guideline for epidemiologic studies implemented in Japan, in 2002, for studies which started before the enforcement of the ethical guideline. This study protocol was first approved by the institutional review board of the National Cancer Center, Tokyo, Japan (Approval number: 2001–021) on the basis of the first ethical guideline for epidemiologic studies, and has been approved annually since 2001.

At baseline survey, participants were asked to answer self-administered questionnaires which inquired about personal and family medical history, as well as smoking habits, alcohol consumption, physical exercise frequency and other lifestyle factors. The participants were also requested to complete a validated food frequency questionnaire [20] that asked about average intake during the previous month of 44 food items (for Cohort I) or 52 food items (for Cohort II). A total of 53,375 men (78%) and 60,086 women (84%) completed and returned the questionnaire. During the same period, 18,159 men and 30,852 women also voluntarily donated 10 mL of venous blood during their health check-ups (acquisition rate: 26% and 43%, respectively). The blood samples were drawn into vacutainer tubes containing heparin, divided into plasma and buffy layers, and preserved at -80°C until analysis.

For the present investigation of colorectal cancer risk, we excluded one PHC area of Cohort I (Katsushika, Tokyo), because cancer incidence data were not available, and limited the study subjects to those who had returned the baseline questionnaire, provided blood samples, and reported no diagnosis of any cancer. Finally, 14,004 men and 24,369 women were followed to the end of 2003. During the follow-up period, changes in residence status, including survival, were determined annually through the residential registry of each PHC area, or for those who had moved out of the study area, through the municipal office of the area to which they had moved to. Among the study subjects, 9.9% moved away, and 0.2% were lost to follow-up during the study period. Cancer incidence was ascertained by active patient notification from major local hospitals in the study area and data linkage with population-based cancer registries, with permission from the local governments responsible for the registries. Death certificate information was used as a supplementary information source, with 5.5% of colorectal cancer cases notified by death certificate. The site of origin and histologic type of colorectal cancer cases were coded using the International Classification of Diseases for Oncology, Third Edition (ICD-O-3), code C180-189, C199 and C209. Up to the end of the study period, we identified 375 cases (196 men and 179 women) of colorectal cancer. All 375 cases were pathologically confirmed as adenocarcinoma. Cases with unknown pathology (18 cases) or non-adenocarcinoma (7 cases) were excluded. Of the 375 cases, 256 subjects had cancer of the colon and 119 had cancer of the rectum. Controls were selected from eligible cohort subjects using the incidence density sampling method. Eligible subjects with no colorectal cancer history at the time the case was diagnosed were matched to cases in terms of age (± 3 years), sex, season of blood sampling (± 3 months), time since last meal (± 4 h), and study location (PHC area), and randomly selected to provide a case:control ratio of 1:2.

### SNP selection and genotyping

To conduct genetic research within the framework of the JPHC Study, we obtained an additional approval from the institutional review board of the National Cancer Center, Tokyo, Japan (Approval number: 2011–044), and provided all eligible subjects who donated a blood sample with the opportunity to refuse participation in the research.

To select tag SNPs in the *VDR* gene, we first nominated 82 candidate SNPs by referring mainly to the International HapMap Project database (Phase 3, release 27, JPT samples), and additionally to a report of *VDR* genetic variations in a Japanese population [12]. Based on the criteria of a minor allele frequency of greater than 10% (in JPT samples) and a pair-wise *r*^*2*^ of greater than 0.90, we finally obtained 29 tag SNPs of the *VDR* gene, including 4 SNPs (*FokI*, *TaqI*, *ApaI*, *Cdx2*) previously reported in the literature. We also included two commonly reported SNPs in the *GC* gene (rs7041 and rs4588).

Genomic DNA was extracted from white blood cells in the buffy coat layer using a FlexiGene DNA kit (Qiagen, Hilden, Germany). Buffy coat samples were not available for all 16 pairs (i.e. 48 subjects) in one PHC area of Cohort II (Suita, Osaka). All but 12 buffy coat samples provided a sufficient amount of genomic DNA to permit genotyping. 46 SNPs, including 29 SNPs of the *VDR* gene and 2 of the *GC* gene, were genotyped on the BioMark Dynamic Array platform (Fluidigm Corporation, South San Francisco, CA, USA) using the TaqMan SNP Genotyping Assays/Drug Metabolism Genotyping Assays (Applied Biosystems, Foster City, CA) at GeneticLab, Hokkaido, Japan. Samples of cases and matched controls were genotyped in the same batch. Plasma 25(OH)D concentrations of the study subjects were previously measured by competitive protein-binding assay at Mitsubishi Kagaku Bio-Clinical Laboratories Inc., Tokyo, Japan using a commercially available reagent (Sigma-Aldrich Co., St Louis, MO, USA). Samples from matched sets were assayed together. The intra-assay coefficient of variation from the quality control samples (n = 9) was 8.4% [8]. All laboratory personnel were blinded with respect to case and control status.

### Statistical analysis

Means, medians, and proportions between cases and controls was compared using the *t*-test, Wilcoxon rank-sum test, and χ^2^ test, respectively. Departure from the Hardy-Weinberg equilibrium was assessed among controls using the χ^2^ test with 1 degree of freedom. The Bonferroni method was used to adjust for multiple correction of the Hardy-Weinberg equilibrium *P* value and the corrected threshold of 0.002 (0.05/29 SNPs) was considered significant. Conditional logistic regression models were used to estimate the odds ratios (OR) and 95% confidence intervals (CI) for the association of *VDR* and *GC* SNPs with colorectal cancer risk. ORs for all SNP/colorectal cancer associations estimated based on assumption of the dominant model are presented, unless otherwise stated. Regression models were adjusted for age, sex, PHC area, fasting time, and season of blood sampling (matching factors), smoking (pack years of smoking), alcohol use (g/week of ethanol), physical activity (once/week, more than once/week), body mass index [(BMI), continuous (kg/m^2^)], and family history of colorectal cancer (yes, no). Further adjustment for dietary folate and intake of red and processed meat did not change the OR appreciably and are therefore not included in the final model.

We also evaluated the influence of *VDR* and *GC* gene polymorphisms on colorectal cancer risk in interaction with plasma vitamin D concentration. For this analysis, all subjects were classified into two categories according to median plasma vitamin D concentration (based on median values among controls), and into two categories according to *VDR* and *GC* genotypes (homozygous for the major allele, or heterozygous and homozygous for the minor allele combined). Effect modification of *VDR* and *GC* polymorphisms on the associations with plasma vitamin D were assessed by the likelihood ratio test with 1 degree of freedom, in which a model which included the interaction terms was compared with one that only included the main effects. Correction for multiple comparisons was done using the “*P* values adjusted for multiple correlated tests” (*P*_ACT_) method developed by Conneely and Boehnke [21]. This method takes account of the correlated nature of the multiple tests performed on proximately located SNPs within a gene. A *P*_ACT_ value of <0.05 was considered statistically significant. All statistical analyses were performed using SAS Software version 9.3 (SAS Institute Inc., Cary, NC, USA) and R Statistical Software version 2.9.1 (R Foundation for Statistical Computing, Vienna, Austria).

## Results

The distribution of some lifestyle and dietary factors among cases and controls is summarized in Table 1. Mean age of the cases and controls was 56.7 and 56.6 years, respectively, and approximately 51% of subjects in both groups were male. The two groups did not statistically significantly differ with regard to BMI, smoking, alcohol use, physical activity or family colorectal cancer history, although the proportion of past/current smokers in the case group appeared to be higher than in controls. The two groups also did not differ with respect to dietary calcium and vitamin D intake and plasma vitamin D concentrations. Dietary vitamin D was weakly correlated with plasma vitamin D. The Spearman partial correlation coefficient after adjustment of age, sex and BMI was 0.08 (*P* = 0.04).

**Table 1:** Baseline characteristics of cases and controls.

Characteristic	Cases (356)	Controls (709)	*P* value
Men, n (%)	183 (51.4)	366 (51.6)	
Age (years), mean (SD)	56.7 (7.3)	56.6 (7.2)	
Body mass index (kg/m^2^), mean (SD)	23.7 (3.0)	23.5 (2.9)	0.31
Past/current smoker, n (%)	154 (43.8)	272 (38.5)	0.10
Past/current alcohol user, n (%)	168 (47.9)	331 (47.8)	0.99
Physical activity (≥1 times/week), n (%)	76 (21.3)	131 (18.5)	0.26
Family colorectal cancer history, n (%)	7 (2.0)	8 (1.1)	0.27
Calcium intake (mg/d), median (IQR)	404 (278–564)	392 (281–542)	0.54
Vitamin D intake (μg/d), median (IQR)	5.1 (3.5–7.3)	5.1 (3.5–6.9)	0.36
Plasma vitamin D (ng/mL), median (IQR)	25.1 (19.9–30.2)	25.1 (20.8–30.5)	0.58

SD, Standard deviation; IQR, interquartile range.

The associations of the *VDR* and *GC* SNPs with colorectal cancer risk are shown in Table 2. The minor allele frequencies of the included SNPs ranged from 0.11 (rs731236, rs10875692) to 0.49 (rs2283342, rs7299460). Genotype frequencies for all *VDR* SNPs (among controls) were in Hardy-Weinberg equilibrium (*P* value > 0.002). Eight *VDR* SNPs, namely rs2254210, rs1540339, rs2107301, rs11168267, rs11574113, rs731236 *(TaqI)*, rs3847987 and rs11574143, the latter 5 of which were located in the 3′ region, were nominally associated with the risk of colorectal cancer. However, none of these associations was statistically significant after correction for multiple testing. Other *VDR* or *GC* SNPs, including the commonly reported *Cdx2*, *FokI* and *ApaI*, were also not associated with colorectal cancer risk. In a sub-group analysis by gender and cancer site (colon or rectal), some of the nominally significant associations observed in the whole population appeared to be limited to males (S1 Table) and to colon cancer (S2 Table), respectively.

**Table 2:** *VDR* and *GC* gene polymorphisms and colorectal cancer risk.

Gene	Variant	Position^a^	Allele^b^	MAF^c^	*P*_*HWE*_^c^	OR (95% CI)^d^	*P*	*P*^*e*^
*VDR*	rs4237856	48338050	A/C	0.22	0.36	0.99 (0.76–1.29)	0.97	1.00
	rs4073729	48337069	G/A	0.21	0.04	1.05 (0.80–1.38)	0.71	1.00
	rs7970314	48308174	G/A	0.48	0.82	1.03 (0.77–1.38)	0.84	1.00
	rs11568820 (*Cdx2*)	48302545	C/T	0.39	0.64	1.00 (0.77–1.31)	0.99	1.00
	rs7299460	48296268	T/C	0.49	0.75	1.03 (0.77–1.39)	0.83	1.00
	rs7136534	48294626	C/T	0.33	0.78	1.05 (0.80–1.36)	0.73	1.00
	rs10875695	48293037	C/A	0.37	0.58	1.01 (0.77–1.31)	0.95	1.00
	rs4334089	48286015	G/A	0.38	0.90	1.08 (0.83–1.41)	0.57	1.00
	rs4760648	48280665	C/T	0.46	0.95	1.11 (0.83–1.47)	0.49	1.00
	rs2853564	48278487	A/G	0.31	0.94	1.27 (0.98–1.66)	0.07	0.70
	rs2238136	48277713	C/T	0.20	0.02	0.81 (0.61–1.07)	0.13	0.86
	rs2254210	48273714	G/A	0.26	0.98	1.32 (1.01–1.73)	0.04	0.53
	rs2228570 (*FokI*)	48272895	G/A	0.38	0.67	1.10 (0.84–1.43)	0.49	1.00
	rs2239186	48269410	A/G	0.48	0.87	0.89 (0.66–1.20)	0.45	1.00
	rs2189480	48263828	T/G	0.38	0.80	1.25 (0.95–1.65)	0.10	0.80
	rs2239179	48257766	T/C	0.24	0.86	1.21 (0.93–1.57)	0.16	0.90
	rs1540339	48257326	T/C	0.27	0.77	1.31 (1.01–1.71)	0.045	0.54
	rs2283342	48255859	G/A	0.49	0.23	1.04 (0.77–1.39)	0.81	1.00
	rs2107301	48255570	A/G	0.28	0.57	1.30 (1.00–1.69)	0.048	0.55
	rs2239182	48255411	T/C	0.23	0.76	1.25 (0.97–1.63)	0.09	0.74
	rs11168267	48251542	G/A	0.18	0.12	0.74 (0.55–0.99)	0.04	0.53
	rs10875692	48239130	C/T	0.11	0.15	1.21 (0.89–1.65)	0.23	0.95
	rs11574113	48238900	G/C	0.20	0.79	0.67 (0.51–0.89)	0.01	0.12
	rs7975232 (*ApaI*)	48238837	C/A	0.31	0.85	0.84 (0.65–1.09)	0.18	0.91
	rs731236 (*TaqI*)	48238757	A/G	0.11	0.56	1.35 (1.00–1.83)	0.048	0.56
	rs3847987	48238068	C/A	0.19	0.76	0.70 (0.52–0.92)	0.01	0.22
	rs11574143	48234917	C/T	0.18	0.20	0.69 (0.51–0.92)	0.01	0.20
	rs7968585	48232093	C/T	0.29	0.21	0.85 (0.66–1.10)	0.22	0.95
	rs12721364	48231430	A/G	0.43	0.81	0.95 (0.73–1.24)	0.71	1.00
*GC*	rs4588	72618323	G/T	0.24	0.96	1.02 (0.79–1.32)	0.86	1.00
	rs7041	72618334	A/C	0.26	0.04	0.97 (0.74–1.27)	0.83	1.00

*VDR* SNPs are ordered by their occurrence in the gene from the 5′ to 3′ end.

^a^Based on hg19.

^b^Major/minor allele.

^c^Minor allele frequency (MAF) and *P*-value for Hardy Weinberg Equilibrium (*P*_HWE_) among controls.

^d^Odds ratios 95% Confidence Interval (CI) based on dominant genetic effect model; all models were adjusted for age, sex, fasting time, season of blood sampling (matching factors), smoking, alcohol use, physical activity, body mass index, and family history of colorectal cancer.

^e^*P* values adjusted for multiple comparisons.

The association of *VDR* and *GC* gene polymorphism with colorectal cancer risk according to plasma vitamin D concentration is shown in Table 3. None of the interaction *P* values was significant after multiple comparison adjustment. Of interest, however, tohe odds ratios for the minor allele carriers for 3 SNPs (rs11574113, rs3847987, and rs11574143), all of which were in the 3′ region and had nominal associations in the SNP/colorectal cancer analysis, showed a significant decrease in the risk of colorectal cancer when they had higher plasma concentrations of vitamin D. Compared to those homozygous for a common allele for SNP, rs11574113, which had low vitamin D, the odds ratios for those homozygous for common alleles with high vitamin D and for those heterozygous and homozygous for minor alleles combined with high vitamin D were 1.09 (95%CI, 0.75–1.58) and 0.61 (95%CI, 0.39–0.97), respectively. The respective values were 1.11 (95%CI, 0.76–1.62) and 0.63 (95%CI, 0.40–0.99) for SNP rs3847987, and 1.13 (95%CI, 0.78–1.64) and 0.60 (95%CI, 0.37–0.95) for SNP rs11574143.

**Table 3:** Association of *VDR* and *GC* gene polymorphisms with colorectal cancer risk according to plasma vitamin D concentration.

Variant		Categories of plasma vitamin D				*P*^c^
		Low (< 25.1 ng/mL)		High (≥ 25.1 ng/mL)		
		N^a^	OR (95% CI)^b^	N^a^	OR (95% CI)^b^	
rs4237856	AA	105/213	1.00 (ref.)	108/210	1.00 (0.67–1.48)	0.74
	CA+CC	73/140	1.04 (0.71–1.52)	70/145	0.95 (0.62–1.47)	
rs4073729	GG	112/228	1.00 (ref.)	106/221	0.95 (0.64–1.40)	0.70
	GA+AA	63/123	1.00 (0.67–1.47)	72/132	1.05 (0.68–1.64)	
rs7970314	AA	44/93	1.00 (ref.)	48/95	0.99 (0.57–1.70)	0.93
	GA+GG	133/260	1.04 (0.68–1.60)	130/259	1.00 (0.62–1.61)	
rs11568820 *(Cdx2)*	CC	67/134	1.00 (ref.)	67/136	0.92 (0.58–1.47)	0.78
	TC+TT	110/219	0.96 (0.66–1.41)	111/218	0.96 (0.63–1.48)	
rs7299460	TT	42/90	1.00 (ref.)	48/93	1.05 (0.61–1.82)	0.70
	TC+CC	136/263	1.10 (0.71–1.68)	130/262	1.03 (0.64–1.65)	
rs7136534	CC	77/158	1.00 (ref.)	77/157	0.96 (0.62–1.50)	1.00
	TC+TT	101/195	1.05 (0.73–1.51)	101/198	1.01 (0.66–1.54)	
rs10875695	CC	70/143	1.00 (ref.)	71/142	0.98 (0.62–1.54)	0.91
	CA+AA	108/210	1.02 (0.70–1.49)	107/213	0.97 (0.64–1.49)	
rs4334089	GG	64/137	1.00 (ref.)	66/136	1.00 (0.63–1.59)	0.84
	GA+AA	114/216	1.11 (0.76–1.63)	112/219	1.05 (0.68–1.62)	
rs4760648	CC	50/104	1.00 (ref.)	45/99	0.88 (0.51–1.53)	0.73
	TC+TT	128/249	1.06 (0.71–1.58)	133/256	1.04 (0.67–1.60)	
rs2853564	AA	82/175	1.00 (ref.)	70/163	0.87 (0.56–1.35)	0.47
	GA+GG	96/178	1.16 (0.80–1.67)	108/192	1.23 (0.80–1.88)	
rs2238136	CC	116/210	1.00 (ref.)	125/231	0.97 (0.66–1.42)	0.90
	TC+TT	62/143	0.82 (0.56–1.20)	53/124	0.77 (0.49–1.20)	
rs2254210	GG	88/201	1.00 (ref.)	84/185	1.01 (0.66–1.55)	0.83
	GA+AA	89/152	1.36 (0.93–1.99)	94/169	1.30 (0.84–2.02)	
rs2228570 *(FokI)*	GG	66/138	1.00 (ref.)	65/135	0.95 (0.59–1.54)	0.96
	GA+AA	112/215	1.09 (0.75–1.59)	113/220	1.05 (0.69–1.61)	
rs2239186	AA	47/94	1.00 (ref.)	57/101	1.07 (0.63–1.84)	0.61
	GA+GG	131/259	0.96 (0.64–1.46)	121/254	0.89 (0.56–1.42)	
rs2189480	TT	62/153	1.00 (ref.)	58/122	1.11 (0.69–1.80)	0.38
	TG+GG	116/200	1.42 (0.97–2.07)	120/233	1.23 (0.81–1.88)	
rs2239179	TT	99/214	1.00 (ref.)	95/201	0.98 (0.65–1.46)	0.85
	TC+CC	79/139	1.24 (0.86–1.78)	83/154	1.15 (0.76–1.75)	
rs1540339	TT	84/197	1.00 (ref.)	88/185	1.05 (0.69–1.60)	0.46
	TC+CC	94/156	1.44 (1.00–2.06)	90/170	1.25 (0.82–1.90)	
rs2283342	GG	45/109	1.00 (ref.)	52/83	1.41 (0.82–2.44)	0.08
	GA+AA	133/244	1.33 (0.88–2.01)	126/272	1.11 (0.70–1.77)	
rs2107301	AA	81/188	1.00 (ref.)	84/181	1.03 (0.67–1.57)	0.62
	GA+GG	97/165	1.38 (0.97–1.98)	94/174	1.25 (0.82–1.91)	
rs2239182	TT	100/216	1.00 (ref.)	93/204	0.95 (0.64–1.42)	0.98
	TC+CC	78/137	1.25 (0.87–1.79)	85/150	1.20 (0.79–1.81)	
rs11168267	GG	127/243	1.00 (ref.)	136/239	1.08 (0.74–1.56)	0.23
	GA+AA	51/110	0.88 (0.59–1.31)	42/116	0.67 (0.42–1.06)	
rs10875692	CC	137/273	1.00 (ref.)	137/287	0.92 (0.64–1.31)	0.45
	TC+TT	41/80	1.08 (0.70–1.66)	41/68	1.25 (0.77–2.04)	
rs11574113	CC	124/228	1.00 (ref.)	134/228	1.09 (0.75–1.58)	0.24
	GC+GG	54/125	0.79 (0.54–1.16)	44/127	0.61 (0.39–0.97)	
rs7975232 (*ApaI*)	CC	85/177	1.00 (ref.)	98/159	1.23 (0.81–1.87)	0.06
	CA+AA	93/176	1.07 (0.74–1.53)	80/196	0.81 (0.53–1.23)	
rs731236 (*TaqI*)	AA	131/292	1.00 (ref.)	133/271	1.05 (0.74–1.50)	0.17
	GA+GG	47/61	1.69 (1.10–2.60)	45/84	1.16 (0.72–1.87)	
rs3847987	CC	124/233	1.00 (ref.)	134/228	1.11 (0.76–1.62)	0.16
	CA+AA	54/120	0.84 (0.57–1.25)	44/127	0.63 (0.40–0.99)	
rs11574143	CC	127/241	1.00 (ref.)	140/235	1.13 (0.78–1.64)	0.09
	TC+TT	51/112	0.87 (0.58–1.29)	38/120	0.60 (0.37–0.95)	
rs7968585	CC	87/187	1.00 (ref.)	104/165	1.28 (0.85–1.94)	0.025
	TC+TT	91/166	1.14 (0.79–1.64)	74/190	0.81 (0.53–1.24)	
rs12721364	AA	57/113	1.00 (ref.)	64/116	1.02 (0.62–1.67)	0.79
	GA+GG	120/240	0.99 (0.67–1.47)	114/238	0.93 (0.60–1.45)	
rs4588	GG	92/182	1.00 (ref.)	108/222	0.95 (0.64–1.42)	0.89
	TG+TT	86/171	1.00 (0.69–1.45)	70/133	0.99 (0.63–1.55)	
rs7041	AA	101/212	1.00 (ref.)	96/170	1.14 (0.75–1.72)	0.20
	CA+CC	77/141	1.15 (0.79–1.67)	82/185	0.92 (0.61–1.40)	

*VDR* SNPs are ordered by their occurrence in the gene from 5′ to 3′ end.

^a^Number of cases/controls.

^b^Odds ratios (OR) and 95% confidence intervals (CI). Adjusted for age, sex, fasting time, and season of blood sampling (matching factors), and smoking, alcohol use, physical activity, body mass index, and family history of colorectal cancer.

^c^*P* values for interaction, unadjusted for multiple comparisons. Interaction *P* value after multiple comparison adjustment was 1.00 for all models except for one associated with rs7968585, which was 0.38.

## Discussion

To our knowledge, this is the first report of *VDR* polymorphisms/colorectal cancer association among Mongoloid Asians that used the systematic tag SNP approach to select variations across the *VDR* gene. In this study, 8 *VDR* SNPs, 5 of which were located in the 3′ region, were nominally associated with the risk of colorectal cancer. Furthermore, of the above 5 3′ region SNPs, the associations for 3 SNPs appeared to be evident only in those with high plasma vitamin D concentration. However, neither the main effect nor the interaction analyses associations were significant after multiple testing adjustment.

Although the interaction effect was not statistically significant, variant allele carriers for 3 SNPs (rs11574113, rs3847987, and rs11574143) showed a significant decrease in the risk of colorectal cancer when they had higher plasma concentrations of vitamin D. Interestingly, these 3 SNPs were all located in the 3′ region and were also nominally associated with colorectal cancer risk in SNP/colorectal cancer analysis. In their comprehensive analysis of vitamin D-related genes in 749 incident prostate cancer cases and 781 controls, Ahn and colleagues reported positive associations between *VDR* tag SNPs in or near the 3′ region and prostate cancer risk among men with low vitamin D, but not with high vitamin D. In particular, this association was stronger for *VDR* variant rs11574143 [22]. In another report from the Cardiovascular Health Study, Levin *et al*. also reported interaction effects of another 3′ *VDR* variant, rs7968585, and low vitamin D in the risk of composite clinical outcomes, including cancer[23]. Although the present study may not be directly comparable to these two studies due to differences in cancer endpoint, vitamin D cut-off and other parameters, these findings might collectively suggest that tag SNPs in and around the 3′ region, in interaction with vitamin D, may be associated with risk of cancer.

The underlying biological basis of the putative interaction effect of the 3′ SNPs, however, is not clear. In fact, the importance of the 3′ end of the *VDR* gene has been examined in relation to the poly(A) microsatellite repeat in the 3′ untranslated region (UTR), the length of which likely determines messenger RNA stability and hence likely affects intracellular levels of VDR [9, 11]. An increase in VDR expression may lead to an enhanced vitamin D endocrine system functioning, given that there is also enough substrate [*i*.*e*. 25(OH)D] for 1,25(OH)_2_D production. This would also explain the association observed for some SNPs in those with higher vitamin D status only. However, it is unknown whether the SNPs under consideration in our study directly influence mRNA stability and VDR expression, or are just related to colorectal cancer through strong linkage disequilibrium with Poly(A) microsatellite repeat polymorphisms or some other functional polymorphisms in the 3′ UTR region. A clear understanding of these associations requires functional studies of the effect of SNPs in the 3′ UTR on VDR expression.

Among other commonly reported 3′ region *VDR* polymorphisms, *BsmI* (rs1544410) polymorphism appears to be associated with decreased risk, with a recent meta-analysis producing a summary risk estimate of 0.89 (95%CI 0.81–0.98) for the *BB* compared to *bb* genotype [5]. The *TaqI* variant (rs731236) was not related to colorectal cancer in three studies [13, 24, 25] but was associated in a US study [26], in which the *tt* genotype was associated with decreased risk of colon cancer (OR 0.6, 95%CI 0.4–1.0). The variant allele for both the *BsmI* and *TaqI* polymorphisms is less frequent in Asians than in Africans and Caucasians [9], as observed in the present and other studies [27, 28]. Although we did not directly measure *BsmI* polymorphism, the OR for *TaqI* polymorphism, which is in high linkage disequilibrium with *BsmI* in the Japanese population (*r*^*2*^ = 0.9), was suggestive of an increased risk, but did not show a statistically significant association. This finding is consistent with an earlier study in a Japanese population [28].

The two *GC* SNPs were not related to the risk of colorectal cancer in our present study. Although tag SNPs in *GC*, including variant rs7041, were related to circulating 25(OH)D concentrations, these SNPs were not associated with the risk of colorectal adenoma in a study by Hibler *et al*. [29]. In studies of colorectal cancer, the *GC* variant rs4588 was not related to colorectal cancer in two studies [30, 31], except for one small study in which homozygosity for the minor allele carried a significantly increased risk [32]. In a Colon Cancer Family Registry based case-control study which included 1,750 sibships, Poynter *et al*. found no evidence for associations between tag SNPs in *GC* and the risk of colorectal cancer, and no evidence for modification of this association by dietary calcium or vitamin D intake [13].

Major strengths of the present study include its use of the comprehensive tag SNP approach to capture common variants across the *VDR* gene. Use of the tag SNP approach has the advantage of greater genetic coverage and statistical power than the traditional approaches to SNP selection [33]. Our inclusion of multiple SNPs from the areas 10 kb upstream and downstream of the actual gene further enhances genetic coverage and perhaps increases the probability of finding markers linked to the causal SNP. Information on all covariates was collected at baseline, i.e. before the diagnosis of cancer, and concerns over information bias are less likely. All cases were pathologically confirmed as adenocarcinoma. We used prediagnostic measurement of serum vitamin D, which reflect both dietary and endogenously formed vitamin D in the circulation and is better marker of circulating vitamin D than the dietary vitamin D measurement alone. Variations in other vitamin D pathway-related genes (eg. *CYP27A1*, *CYP2R1*, *CYP27B1*, *CYP24A1*, *RXRA)* might also be associated with the risk of colorectal cancer, and our inability to evaluate these variants therefore constitutes a weakness of the study. Plasma vitamin D concentration was also measured once only, and therefore might not represent long-term variation in vitamin D concentration. However, previous studies have shown a fairly good correlation among vitamin D concentrations measured at several-year intervals [34, 35] indicating that a single vitamin D measurement is reasonably representative. Finally, another limitation of the study was its relatively small sample size and limited power to detect moderate associations. With a sample size of about 350 cases and 700 matched-control subjects, the power (two-sided unadjusted α = 0.05) to detect an OR in the range of 1.2 to 1.4 (in the dominant model) was between 21% to 59% for a variant with a low minor allele (rs731236), while the power to detect an OR in the range of 0.8 to 1.4 was from 34% to 57% for a variant with a more common minor allele (rs12721364). Therefore, the current sample size was underpowered to detect SNP/colorectal cancer associations of the order of magnitude as found in this study.

In summary, the findings of this nested case-control study among a Japanese population provides only limited support for an association between common variations in the *VDR* gene and colorectal cancer risk. A better understanding of these associations requires further studies with larger sample sizes and other vitamin D pathway genes.

## Appendix

Members of the Japan Public Health Center-based Prospective Study (JPHC Study, principal investigator: S. Tsugane) Group are: S. Tsugane, N. Sawada, M. Iwasaki, S. Sasazuki, T. Yamaji, T. Shimazu and T. Hanaoka, National Cancer Center, Tokyo; J. Ogata, S. Baba, T. Mannami, A. Okayama, and Y. Kokubo, National Cerebral and Cardiovascular Center, Osaka; K. Miyakawa, F. Saito, A. Koizumi, Y. Sano, I. Hashimoto, T. Ikuta, Y. Tanaba, H. Sato, Y. Roppongi, T. Takashima and H. Suzuki, Iwate Prefectural Ninohe Public Health Center, Iwate; Y. Miyajima, N. Suzuki, S. Nagasawa, Y. Furusugi, N. Nagai, Y. Ito, S. Komatsu and T. Minamizono, Akita Prefectural Yokote Public Health Center, Akita; H. Sanada, Y. Hatayama, F. Kobayashi, H. Uchino, Y. Shirai, T. Kondo, R. Sasaki, Y. Watanabe, Y. Miyagawa, Y. Kobayashi, M. Machida, K. Kobayashi and M. Tsukada, Nagano Prefectural Saku Public Health Center, Nagano; Y. Kishimoto, E. Takara, T. Fukuyama, M. Kinjo, M. Irei, and H. Sakiyama, Okinawa Prefectural Chubu Public Health Center, Okinawa; K. Imoto, H. Yazawa, T. Seo, A. Seiko, F. Ito, F. Shoji and R. Saito, Katsushika Public Health Center, Tokyo; A. Murata, K. Minato, K. Motegi, T. Fujieda and S. Yamato, Ibaraki Prefectural Mito Public Health Center, Ibaraki; K. Matsui, T. Abe, M. Katagiri, M. Suzuki, K. and Matsui, Niigata Prefectural Kashiwazaki and Nagaoka Public Health Center, Niigata; M. Doi, A. Terao, Y. Ishikawa, and T. Tagami, Kochi Prefectural Chuo-higashi Public Health Center, Kochi; H. Sueta, H. Doi, M. Urata, N. Okamoto, F. Ide, H. Goto and R Fujita, Nagasaki Prefectural Kamigoto Public Health Center, Nagasaki; H. Sakiyama, N. Onga, H. Takaesu, M. Uehara, T. Nakasone and M. Yamakawa, Okinawa Prefectural Miyako Public Health Center, Okinawa; F. Horii, I. Asano, H. Yamaguchi, K. Aoki, S. Maruyama, M. Ichii, and M. Takano, Osaka Prefectural Suita Public Health Center, Osaka; Y. Tsubono, Tohoku University, Miyagi; K. Suzuki, Research Institute for Brain and Blood Vessels Akita, Akita; Y. Honda, K. Yamagishi, S. Sakurai and N. Tsuchiya, University of Tsukuba, Ibaraki; M. Kabuto, National Institute for Environmental Studies, Ibaraki; M. Yamaguchi, Y. Matsumura, S. Sasaki, and S. Watanabe, National Institute of Health and Nutrition, Tokyo; M. Akabane, Tokyo University of Agriculture, Tokyo; T. Kadowaki and M. Inoue, The University of Tokyo, Tokyo; M. Noda and T. Mizoue, National Center for Global Health and Medicine, Tokyo; Y. Kawaguchi, Tokyo Medical and Dental University, Tokyo; Y. Takashima and Y. Yoshida, Kyorin University, Tokyo; K. Nakamura and R. Takachi, Niigata University, Niigata; J. Ishihara, Sagami Women’s University, Kanagawa; S. Matsushima and S. Natsukawa, Saku General Hospital, Nagano; H. Shimizu, Sakihae Institute, Gifu; H. Sugimura, Hamamatsu University School of Medicine, Shizuoka; S. Tominaga, Aichi Cancer Center, Aichi; N. Hamajima, Nagoya University, Aichi; H. Iso and T. Sobue, Osaka University, Osaka; M. Iida, W. Ajiki, and A. Ioka, Osaka Medical Center for Cancer and Cardiovascular Disease, Osaka; S. Sato, Chiba Prefectural Institute of Public Health, Chiba; E. Maruyama, Kobe University, Hyogo; M. Konishi, K. Okada, and I. Saito, Ehime University, Ehime; N. Yasuda, Kochi University, Kochi; S. Kono, Kyushu University, Fukuoka; S. Akiba, Kagoshima University, Kagoshima; T. Isobe, Keio University; Y. Sato, Tokyo Gakugei University.

## Supporting Information

S1 Table *VDR* and *GC* gene polymorphisms and colorectal cancer risk in men and women. (DOCX)

S2 Table *VDR* and *GC* gene polymorphisms and colon and rectal cancer risk. (DOCX)

S3 Table *VDR* and *GC* gene polymorphisms and colorectal cancer risk (Additive and Recessive models). (DOCX)

S4 Table *VDR* and *GC* gene polymorphisms and colorectal cancer risk by plasma vitamin D concentration. (DOCX)
