# Supplementary material for: Vitamin D Receptor Gene Polymorphism and the Risk of Colorectal Cancer: A Nested Case-Control Study
Source: PLoS One. 2016 Oct 13;11(10):e0164648. doi: 10.1371/journal.pone.0164648 (PMC5063384; doi:10.1371/journal.pone.0164648)
Supplement: S1 Table — (DOCX) [file pone.0164648.s001.docx]

**S1 Table. *VDR* and *GC* gene polymorphisms and colorectal cancer risk in men and women.**

| Gene | Variants | Men | | |  | Women | | |
| --- | --- | --- | --- | --- | --- | --- | --- | --- |
|  |  | OR (95% CI)^a^ | *P*^b^ | *P*^c^ |  | OR (95% CI)^a^ | *P*^b^ | *P*^c^ |
| *VDR* | rs4237856 | 0.79 (0.54-1.15) | 0.22 | 0.95 |  | 1.20 (0.82-1.74) | 0.34 | 1.00 |
|  | rs4073729 | 1.20 (0.81-1.77) | 0.36 | 0.98 |  | 0.93 (0.63-1.36) | 0.70 | 1.00 |
|  | rs7970314 | 0.87 (0.58-1.32) | 0.52 | 1.00 |  | 1.23 (0.80-1.88) | 0.35 | 1.00 |
|  | rs11568820 | 1.08 (0.74-1.57) | 0.70 | 1.00 |  | 0.94 (0.64-1.37) | 0.75 | 1.00 |
|  | rs7299460 | 0.91 (0.60-1.37) | 0.65 | 1.00 |  | 1.19 (0.77-1.83) | 0.43 | 1.00 |
|  | rs7136534 | 1.17 (0.80-1.70) | 0.42 | 0.98 |  | 0.94 (0.65-1.37) | 0.75 | 1.00 |
|  | rs10875695 | 1.18 (0.81-1.72) | 0.38 | 0.98 |  | 0.85 (0.58-1.24) | 0.40 | 1.00 |
|  | rs4334089 | 1.14 (0.78-1.66) | 0.51 | 1.00 |  | 1.03 (0.71-1.51) | 0.86 | 1.00 |
|  | rs4760648 | 1.24 (0.83-1.85) | 0.29 | 0.97 |  | 1.00 (0.66-1.51) | 0.98 | 1.00 |
|  | rs2853564 | 1.35 (0.93-1.96) | 0.12 | 0.83 |  | 1.17 (0.80-1.71) | 0.43 | 1.00 |
|  | rs2238136 | 0.69 (0.46-1.03) | 0.07 | 0.68 |  | 0.95 (0.64-1.39) | 0.78 | 1.00 |
|  | rs2254210 | 1.29 (0.89-1.86) | 0.18 | 0.93 |  | 1.32 (0.88-1.97) | 0.18 | 0.95 |
|  | rs2228570 | 0.86 (0.59-1.25) | 0.43 | 0.98 |  | 1.39 (0.94-2.05) | 0.10 | 0.82 |
|  | rs2239186 | 0.90 (0.59-1.38) | 0.63 | 1.00 |  | 0.88 (0.58-1.35) | 0.56 | 1.00 |
|  | rs2189480 | 1.49 (1.01-2.20) | 0.05 | 0.57 |  | 1.09 (0.74-1.60) | 0.67 | 1.00 |
|  | rs2239179 | 1.24 (0.85-1.79) | 0.26 | 0.97 |  | 1.22 (0.83-1.80) | 0.31 | 0.99 |
|  | rs1540339 | 1.28 (0.89-1.86) | 0.19 | 0.93 |  | 1.40 (0.95-2.06) | 0.09 | 0.80 |
|  | rs2283342 | 0.84 (0.56-1.26) | 0.40 | 0.98 |  | 1.30 (0.84-2.02) | 0.24 | 0.98 |
|  | rs2107301 | 1.23 (0.85-1.77) | 0.27 | 0.97 |  | 1.45 (0.99-2.12) | 0.06 | 0.65 |
|  | rs2239182 | 1.32 (0.92-1.90) | 0.14 | 0.86 |  | 1.23 (0.84-1.79) | 0.29 | 0.99 |
|  | rs11168267 | 0.57 (0.37-0.86) | 0.01 | 0.15 |  | 0.91 (0.60-1.38) | 0.65 | 1.00 |
|  | rs10875692 | 0.96 (0.62-1.51) | 0.87 | 1.00 |  | 1.51 (0.97-2.35) | 0.07 | 0.71 |
|  | rs11574113 | 0.53 (0.35-0.80) | 0.002 | 0.05 |  | 0.80 (0.54-1.20) | 0.29 | 0.99 |
|  | rs7975232 | 0.73 (0.51-1.06) | 0.10 | 0.79 |  | 0.92 (0.63-1.32) | 0.64 | 1.00 |
|  | rs731236 | 1.46 (0.96-2.23) | 0.08 | 0.72 |  | 1.27 (0.82-1.96) | 0.28 | 0.99 |
|  | rs3847987 | 0.53 (0.35-0.80) | 0.002 | 0.05 |  | 0.87 (0.58-1.31) | 0.49 | 1.00 |
|  | rs11574143 | 0.5 (0.32-0.76) | 0.001 | 0.03 |  | 0.90 (0.59-1.36) | 0.61 | 1.00 |
|  | rs7968585 | 0.72 (0.49-1.04) | 0.08 | 0.72 |  | 0.97 (0.67-1.40) | 0.87 | 1.00 |
|  | rs12721364 | 0.83 (0.57-1.20) | 0.32 | 0.98 |  | 1.07 (0.72-1.60) | 0.73 | 1.00 |
| *GC* | rs4588 | 1.05 (0.73-1.50) | 0.79 | 1.00 |  | 1.02 (0.70-1.49) | 0.92 | 1.00 |
|  | rs7041 | 0.93 (0.63-1.38) | 0.72 | 1.00 |  | 1.00 (0.68-1.47) | 0.99 | 1.00 |

^a^Odds ratios (95% Confidence Interval) based on dominant genetic effect model; adjusted for smoking, alcohol use, physical activity, BMI, and family history of colorectal cancer. *^b^* unadjusted *P* value. *^c^P* values adjusted for multiple comparisons.
