# Supplementary material for: Vitamin D Receptor Gene Polymorphism and the Risk of Colorectal Cancer: A Nested Case-Control Study
Source: PLoS One. 2016 Oct 13;11(10):e0164648. doi: 10.1371/journal.pone.0164648 (PMC5063384; doi:10.1371/journal.pone.0164648)
Supplement: S2 Table — (DOCX) [file pone.0164648.s002.docx]

**S2 Table. *VDR* and *GC* gene polymorphisms and colon and rectal cancer risk.**

| Gene | Variants | Colon Cancer | | |  | Rectal Cancer | | |
| --- | --- | --- | --- | --- | --- | --- | --- | --- |
|  |  | OR (95% CI)^a^ | *P^b^* | *P^c^* |  | OR (95% CI)^a^ | *P^b^* | *P^c^* |
| *VDR* | rs4237856 | 1.03 (0.75-1.43) | 0.85 | 1.00 |  | 0.93 (0.58-1.50) | 0.78 | 1.00 |
|  | rs4073729 | 0.98 (0.71-1.36) | 0.90 | 1.00 |  | 1.25 (0.76-2.05) | 0.39 | 1.00 |
|  | rs7970314 | 1.04 (0.72-1.49) | 0.84 | 1.00 |  | 1.02 (0.61-1.72) | 0.93 | 1.00 |
|  | rs11568820 | 0.96 (0.70-1.31) | 0.79 | 1.00 |  | 1.13 (0.69-1.85) | 0.64 | 1.00 |
|  | rs7299460 | 1.04 (0.72-1.49) | 0.84 | 1.00 |  | 1.03 (0.61-1.73) | 0.92 | 1.00 |
|  | rs7136534 | 1.01 (0.74-1.39) | 0.93 | 1.00 |  | 1.13 (0.70-1.82) | 0.62 | 1.00 |
|  | rs10875695 | 0.94 (0.68-1.28) | 0.69 | 1.00 |  | 1.21 (0.74-2.00) | 0.45 | 1.00 |
|  | rs4334089 | 1.03 (0.75-1.41) | 0.87 | 1.00 |  | 1.22 (0.74-2.02) | 0.44 | 1.00 |
|  | rs4760648 | 1.10 (0.78-1.55) | 0.59 | 1.00 |  | 1.15 (0.69-1.93) | 0.59 | 1.00 |
|  | rs2853564 | 1.23 (0.89-1.70) | 0.21 | 0.97 |  | 1.36 (0.85-2.18) | 0.20 | 0.98 |
|  | rs2238136 | 0.84 (0.60-1.18) | 0.33 | 0.99 |  | 0.75 (0.47-1.22) | 0.25 | 0.99 |
|  | rs2254210 | 1.35 (0.98-1.87) | 0.07 | 0.70 |  | 1.28 (0.79-2.08) | 0.31 | 1.00 |
|  | rs2228570 | 1.13 (0.82-1.57) | 0.45 | 1.00 |  | 1.03 (0.64-1.64) | 0.91 | 1.00 |
|  | rs2239186 | 0.94 (0.65-1.35) | 0.72 | 1.00 |  | 0.81 (0.49-1.36) | 0.43 | 1.00 |
|  | rs2189480 | 1.38 (0.99-1.93) | 0.05 | 0.62 |  | 1.01 (0.62-1.64) | 0.96 | 1.00 |
|  | rs2239179 | 1.23 (0.89-1.70) | 0.21 | 0.96 |  | 1.16 (0.73-1.85) | 0.54 | 1.00 |
|  | rs1540339 | 1.34 (0.97-1.85) | 0.07 | 0.69 |  | 1.24 (0.78-1.97) | 0.37 | 1.00 |
|  | rs2283342 | 1.11 (0.78-1.59) | 0.56 | 1.00 |  | 0.89 (0.52-1.53) | 0.68 | 1.00 |
|  | rs2107301 | 1.35 (0.98-1.87) | 0.06 | 0.67 |  | 1.19 (0.76-1.88) | 0.44 | 1.00 |
|  | rs2239182 | 1.26 (0.92-1.74) | 0.15 | 0.91 |  | 1.24 (0.79-1.94) | 0.35 | 1.00 |
|  | rs11168267 | 0.68 (0.47-0.97) | 0.03 | 0.46 |  | 0.87 (0.53-1.45) | 0.60 | 1.00 |
|  | rs10875692 | 1.16 (0.80-1.67) | 0.43 | 1.00 |  | 1.42 (0.80-2.53) | 0.23 | 0.99 |
|  | rs11574113 | 0.62 (0.44-0.88) | 0.01 | 0.15 |  | 0.76 (0.46-1.26) | 0.28 | 0.99 |
|  | rs7975232 | 0.85 (0.62-1.16) | 0.30 | 0.99 |  | 0.81 (0.51-1.27) | 0.36 | 1.00 |
|  | rs731236 | 1.41 (0.97-2.05) | 0.07 | 0.69 |  | 1.25 (0.75-2.11) | 0.39 | 1.00 |
|  | rs3847987 | 0.63 (0.45-0.90) | 0.01 | 0.19 |  | 0.83 (0.50-1.37) | 0.46 | 1.00 |
|  | rs11574143 | 0.62 (0.43-0.89) | 0.01 | 0.16 |  | 0.83 (0.50-1.38) | 0.48 | 1.00 |
|  | rs7968585 | 0.82 (0.60-1.13) | 0.23 | 0.97 |  | 0.90 (0.58-1.42) | 0.66 | 1.00 |
|  | rs12721364 | 0.98 (0.70-1.36) | 0.90 | 1.00 |  | 0.89 (0.56-1.42) | 0.63 | 1.00 |
| *GC* | rs4588 | 0.95 (0.70-1.30) | 0.77 | 1.00 |  | 1.16 (0.73-1.83) | 0.53 | 1.00 |
|  | rs7041 | 1.00 (0.72-1.39) | 0.99 | 1.00 |  | 0.96 (0.59-1.55) | 0.85 | 1.00 |

^a^Odds ratios (95% Confidence Interval) based on dominant genetic effect model; adjusted for smoking, alcohol use, physical activity, BMI, and family history of colorectal cancer. *^b^* unadjusted *P* value. *^c^P* values adjusted for multiple comparisons.
