# Supplementary material for: Vitamin D Receptor Gene Polymorphism and the Risk of Colorectal Cancer: A Nested Case-Control Study
Source: PLoS One. 2016 Oct 13;11(10):e0164648. doi: 10.1371/journal.pone.0164648 (PMC5063384; doi:10.1371/journal.pone.0164648)
Supplement: S3 Table — (DOCX) [file pone.0164648.s003.docx]

**S3 Table. *VDR* and *GC* gene polymorphisms and colorectal cancer risk (Additive and Recessive models).**

| *Gene* | Variant | Additive model | | |  | Recessive model | | |
| --- | --- | --- | --- | --- | --- | --- | --- | --- |
|  |  | OR (95% CI)^a^ | *P^b^* | *P^c^* |  | OR (95% CI)^a^ | *P^b^* | *P^c^* |
| *VDR* | rs4237856 | 0.99 (0.79-1.23) | 0.91 | 1.00 |  | 0.93 (0.49-1.77) | 0.82 | 1.00 |
|  | rs4073729 | 1.07 (0.86-1.33) | 0.55 | 1.00 |  | 1.25 (0.72-2.17) | 0.43 | 1.00 |
|  | rs7970314 | 0.94 (0.78-1.14) | 0.53 | 1.00 |  | 0.81 (0.59-1.12) | 0.21 | 0.98 |
|  | rs11568820 | 0.94 (0.78-1.14) | 0.53 | 1.00 |  | 0.79 (0.54-1.15) | 0.22 | 0.99 |
|  | rs7299460 | 0.93 (0.77-1.12) | 0.43 | 1.00 |  | 0.78 (0.57-1.07) | 0.13 | 0.93 |
|  | rs7136534 | 0.97 (0.80-1.19) | 0.79 | 1.00 |  | 0.78 (0.50-1.21) | 0.27 | 0.99 |
|  | rs10875695 | 0.93 (0.77-1.13) | 0.47 | 1.00 |  | 0.74 (0.49-1.10) | 0.13 | 0.93 |
|  | rs4334089 | 0.99 (0.82-1.19) | 0.90 | 1.00 |  | 0.81 (0.55-1.19) | 0.29 | 1.00 |
|  | rs4760648 | 1.00 (0.83-1.20) | 0.98 | 1.00 |  | 0.88 (0.63-1.21) | 0.42 | 1.00 |
|  | rs2853564 | 1.14 (0.94-1.40) | 0.18 | 0.91 |  | 0.99 (0.64-1.52) | 0.95 | 1.00 |
|  | rs2238136 | 0.82 (0.64-1.05) | 0.12 | 0.82 |  | 0.75 (0.31-1.79) | 0.51 | 1.00 |
|  | rs2254210 | 1.19 (0.96-1.47) | 0.11 | 0.81 |  | 0.97 (0.58-1.62) | 0.90 | 1.00 |
|  | rs2228570 | 1.10 (0.92-1.32) | 0.29 | 0.98 |  | 1.22 (0.86-1.71) | 0.26 | 0.99 |
|  | rs2239186 | 0.96 (0.79-1.15) | 0.64 | 1.00 |  | 1.00 (0.73-1.37) | 0.99 | 1.00 |
|  | rs2189480 | 1.21 (1.00-1.45) | 0.048 | 0.54 |  | 1.35 (0.95-1.91) | 0.10 | 0.87 |
|  | rs2239179 | 1.17 (0.95-1.45) | 0.14 | 0.86 |  | 1.24 (0.74-2.11) | 0.42 | 1.00 |
|  | rs1540339 | 1.25 (1.02-1.53) | 0.03 | 0.43 |  | 1.36 (0.86-2.17) | 0.19 | 0.98 |
|  | rs2283342 | 1.06 (0.89-1.28) | 0.50 | 1.00 |  | 1.14 (0.85-1.54) | 0.38 | 1.00 |
|  | rs2107301 | 1.25 (1.02-1.52) | 0.03 | 0.40 |  | 1.39 (0.90-2.15) | 0.13 | 0.93 |
|  | rs2239182 | 1.22 (0.99-1.51) | 0.07 | 0.63 |  | 1.35 (0.80-2.29) | 0.26 | 0.99 |
|  | rs11168267 | 0.77 (0.60-0.99) | 0.04 | 0.52 |  | 0.71 (0.33-1.51) | 0.37 | 1.00 |
|  | rs10875692 | 1.15 (0.87-1.52) | 0.34 | 0.99 |  | 0.73 (0.23-2.30) | 0.60 | 1.00 |
|  | rs11574113 | 0.73 (0.57-0.93) | 0.01 | 0.19 |  | 0.79 (0.38-1.64) | 0.53 | 1.00 |
|  | rs7975232 | 0.92 (0.76-1.12) | 0.43 | 1.00 |  | 1.12 (0.72-1.75) | 0.61 | 1.00 |
|  | rs731236 | 1.30 (0.99-1.71) | 0.06 | 0.58 |  | 1.28 (0.48-3.42) | 0.62 | 1.00 |
|  | rs3847987 | 0.75 (0.59-0.96) | 0.02 | 0.32 |  | 0.83 (0.40-1.72) | 0.61 | 1.00 |
|  | rs11574143 | 0.75 (0.58-0.97) | 0.03 | 0.42 |  | 1.18 (0.52-2.69) | 0.69 | 1.00 |
|  | rs7968585 | 0.96 (0.78-1.17) | 0.68 | 1.00 |  | 1.38 (0.86-2.22) | 0.18 | 0.98 |
|  | rs12721364 | 1.00 (0.83-1.19) | 0.96 | 1.00 |  | 1.06 (0.77-1.48) | 0.71 | 1.00 |
| *GC* | rs4588 | 1.00 (0.81-1.23) | 0.97 | 1.00 |  | 0.88 (0.51-1.54) | 0.66 | 1.00 |
|  | rs7041 | 1.00 (0.80-1.25) | 0.99 | 1.00 |  | 1.14 (0.64-2.03) | 0.66 | 1.00 |

^a^Adjusted for smoking, alcohol use, physical activity, BMI, and family history of colorectal cancer. *^b^* unadjusted *P* value. *^c^P* values adjusted for multiple comparisons.
