# Supplementary material for: Vitamin D Receptor Gene Polymorphism and the Risk of Colorectal Cancer: A Nested Case-Control Study
Source: PLoS One. 2016 Oct 13;11(10):e0164648. doi: 10.1371/journal.pone.0164648 (PMC5063384; doi:10.1371/journal.pone.0164648)
Supplement: S4 Table — (DOCX) [file pone.0164648.s004.docx]

**S4 Table. *VDR* and *GC* gene polymorphisms and colorectal cancer risk by plasma vitamin D concentration.**

| *Gene* | Variants | Plasma Vitamin D [OR (95 %CI)^a^] | | | *P^b^* |
| --- | --- | --- | --- | --- | --- |
|  |  | Low (< 25.1 ng/mL) |  | High (≥ 25.1 ng/mL) |  |
| *VDR* | rs4237856 | 1.04 (0.71-1.52) |  | 0.95 (0.66-1.38) | 0.74 |
|  | rs4073729 | 1.00 (0.67-1.47) |  | 1.11 (0.75-1.64) | 0.70 |
|  | rs7970314 | 1.04 (0.68-1.60) |  | 1.02 (0.67-1.53) | 0.93 |
|  | rs11568820 | 0.96 (0.66-1.41) |  | 1.04 (0.71-1.52) | 0.78 |
|  | rs7299460 | 1.10 (0.71-1.68) |  | 0.98 (0.65-1.47) | 0.70 |
|  | rs7136534 | 1.05 (0.73-1.51) |  | 1.05 (0.72-1.52) | 0.99 |
|  | rs10875695 | 1.02 (0.70-1.49) |  | 0.99 (0.68-1.45) | 0.92 |
|  | rs4334089 | 1.11 (0.76-1.63) |  | 1.05 (0.72-1.53) | 0.84 |
|  | rs4760648 | 1.06 (0.71-1.58) |  | 1.17 (0.77-1.78) | 0.73 |
|  | rs2853564 | 1.16 (0.80-1.67) |  | 1.41 (0.96-2.07) | 0.47 |
|  | rs2238136 | 0.82 (0.56-1.20) |  | 0.79 (0.53-1.18) | 0.90 |
|  | rs2254210 | 1.36 (0.93-1.99) |  | 1.29 (0.88-1.88) | 0.83 |
|  | rs2228570 | 1.09 (0.75-1.59) |  | 1.11 (0.75-1.63) | 0.96 |
|  | rs2239186 | 0.96 (0.64-1.46) |  | 0.83 (0.54-1.25) | 0.61 |
|  | rs2189480 | 1.42 (0.97-2.07) |  | 1.11 (0.75-1.63) | 0.38 |
|  | rs2239179 | 1.24 (0.86-1.78) |  | 1.18 (0.81-1.72) | 0.85 |
|  | rs1540339 | 1.44 (1.00-2.06) |  | 1.19 (0.81-1.73) | 0.46 |
|  | rs2283342 | 1.33 (0.88-2.01) |  | 0.79 (0.51-1.20) | 0.08 |
|  | rs2107301 | 1.38 (0.97-1.98) |  | 1.22 (0.84-1.76) | 0.62 |
|  | rs2239182 | 1.25 (0.87-1.79) |  | 1.26 (0.87-1.83) | 0.98 |
|  | rs11168267 | 0.88 (0.59-1.31) |  | 0.62 (0.41-0.94) | 0.23 |
|  | rs10875692 | 1.08 (0.70-1.66) |  | 1.37 (0.88-2.13) | 0.45 |
|  | rs11574113 | 0.79 (0.54-1.16) |  | 0.56 (0.37-0.85) | 0.24 |
|  | rs7975232 | 1.07 (0.74-1.53) |  | 0.65 (0.45-0.95) | 0.06 |
|  | rs731236 | 1.69 (1.10-2.60) |  | 1.10 (0.72-1.68) | 0.17 |
|  | rs3847987 | 0.84 (0.57-1.25) |  | 0.56 (0.37-0.85) | 0.16 |
|  | rs11574143 | 0.87 (0.58-1.29) |  | 0.53 (0.34-0.81) | 0.09 |
|  | rs7968585 | 1.14 (0.79-1.64) |  | 0.63 (0.44-0.91) | 0.03 |
|  | rs12721364 | 0.99 (0.67-1.47) |  | 0.92 (0.63-1.33) | 0.79 |
| *GC* | rs4588 | 1.00 (0.69-1.45) |  | 1.04 (0.71-1.52) | 0.89 |
|  | rs7041 | 1.15 (0.79-1.67) |  | 0.81 (0.55-1.20) | 0.20 |

^a^Odds ratios (95% Confidence Interval) based on dominant genetic effect model; adjusted for smoking, alcohol use, physical activity, BMI, and family history of colorectal cancer. *^b^P* value for interaction, not adjusted for multiple comparisons.
